# Molecular Prevalence of *Leishmania infantum* Infection from Oral Swabs Collected from Dogs in Region of Northwestern Spain

**DOI:** 10.3390/pathogens14060569

**Published:** 2025-06-06

**Authors:** Javier Merino-Goyenechea, Elora Valderas-García, Verónica Castilla Gómez de Agüero, Rafael Balaña-Fouce, María Martínez-Valladares

**Affiliations:** 1 Departamento de Ciencias Biomédicas, Instituto de Biomedicina (IBIOMED), Facultad de Veterinaria, Universidad de León, 24071 León, Spain; luisjaviermerino4@gmail.com (J.M.-G.); rbalf@unileon.es (R.B.-F.); 2 Instituto de Ganadería de Montaña, Departamento de Sanidad Animal, CSIC-Universidad de León, Grulleros, 24346 León, Spain; evalg@unileon.es; 3 Departamento de Ciencias Biomédicas, Instituto de Parasitología y Biomedicina “López—Neyra”, Armilla, 18016 Granada, Spain; veronica.castilla@ipb.csic.es

**Keywords:** dogs, *Leishmania*, canine leishmaniasis, qPCR, prevalence, northwest Spain

## Abstract

Leishmaniasis is a serious zoonotic parasitic disease caused by the protist *Leishmania infantum* and transmitted by phlebotomine sandflies in the countries of the Mediterranean basin. Dogs are the species most susceptible to the disease and serve as a reservoir for transmission to humans, making the Iberian Peninsula an endemic region for this infection. Although the regions close to the Mediterranean coast are the most prevalent regions of leishmaniasis in Spain, climatic factors are favouring the expansion of the vectors to more northern latitudes, where the disease was hardly known decades ago. This paper presents a prevalence study of *L. infantum* infection in the province of Zamora (northwestern Spain) using a non-invasive sample from canine buccal swabs and an innovative qPCR method to determine the etiologic agent. The parasite load of 151 randomly selected dogs from different points of the province was analysed during the period 2021–2022, with an estimated prevalence of 30%. In addition, the most common clinical signs of leishmaniasis in the dogs are described, and intrinsic factors associated with the nature of the dogs—such as sex, size, age as well as other factors related to the habitat in which they live and their geographical location—which could favour the disease, are evaluated.

## 1. Introduction

Canine leishmaniasis (CanL) is a vector-borne disease caused by the protozoan parasite *Leishmania infantum* and transmitted by sandflies of the genus *Phlebotomus* in the Mediterranean basin [1,2,3]. *L. infantum* is considered endemic in this region but is also present in South America and central and southwestern Asia [4]. Domestic dogs are both primary reservoirs and definitive hosts of *L. infantum* in urban areas [5], but there is increasing evidence of leishmaniasis in wildlife, including wild canids, rodents, mustelids, chiroptera, and others [6]. The presence of reservoirs of the pathogen and the zoonotic nature of transmission constitutes a threat to the health of humans, who are definitive hosts and in whom *L. infantum* causes severe and potentially fatal infection [7]. In fact, the WHO considers the various forms of leishmaniasis as one of the three neglected tropical diseases (NTDs) caused by protozoa [8]. Globally, an estimated 3.8–4.5 million people are infected with *Leishmania* spp. [9] and more than 5000 deaths are reported each year, mostly in eastern countries of sub-Saharan Africa [10].

The clinical presentation of CanL can range widely, from infection without clinical signs, to cutaneous and/or mucosal lesions to visceral involvement. Visceral lesions usually include peripheral lymphadenopathy, splenomegaly, hepatomegaly and glomerulonephritis. Cutaneous manifestations include alopecia, dermatitis, onychogryphosis, skin nodules or mucocutaneous lesions [11]. In humans, however, visceral symptoms prevail over skin lesions, which can be fatal without adequate pharmacological treatment [7]. Due to their physical proximity, often sharing the same living environment, and their familiar character, dogs constitute an epidemiological threat to humans that should be prevented. For this reason, an accurate diagnosis is essential to implement control measures and to prevent the occurrence of clinical signs by allowing rapid and suitable treatment. Molecular-based methodologies, such as the polymerase chain reaction (PCR), are sensitive and specific techniques that allow detection and identification of the parasite not only in active infections. Within PCR-based methods, quantitative PCR (qPCR) addresses several limitations inherent to standard PCR techniques, such as reducing the risk of contamination, quantifying the parasite load and improving the limit of detection [12,13].

*L. infantum* MON1 zymodeme is considered the main responsible for CanL in Mediterranean countries [14]. According to a seroprevalence study conducted by Bayer in collaboration with Idexx in 2012, the overall prevalence of CanL in Spain was 15.7%, but varied according to geographical area [15]. Thus, the seroprevalence of CanL cases in the northwest of the country barely reaches 2%, but can range from 31.6% to more than 40% in the southern provinces of Andalusia and the southeast of the Mediterranean coast, respectively [16,17]. Gálvez and co-workers classified the prevalence of CanL in the Iberian Peninsula into four epidemiological regions: (i) leishmaniasis-free, Canary Islands; (ii) low endemicity, Cantabrian coast, Galicia—except Ourense province—Navarra, La Rioja and north of Castilla y León; (iii) endemic, Meseta Central, Badajoz and north of the Mediterranean coast—except Girona; (iv) hyperendemic, Andalusia, Valencian Community and Balearic Islands [18].

Given the scarcity of data on the prevalence of CanL in dogs in northwestern Spain, we have studied the presence of *L. infantum* in buccal swab samples from dogs in the province of Zamora by qPCR analysis during the years 2021–2022 to contribute to the current picture of this disease in Spain.

## 2. Materials and Methods

### 2.1. Animals Included in the Study and Geographical Setting

**Figure 1:**
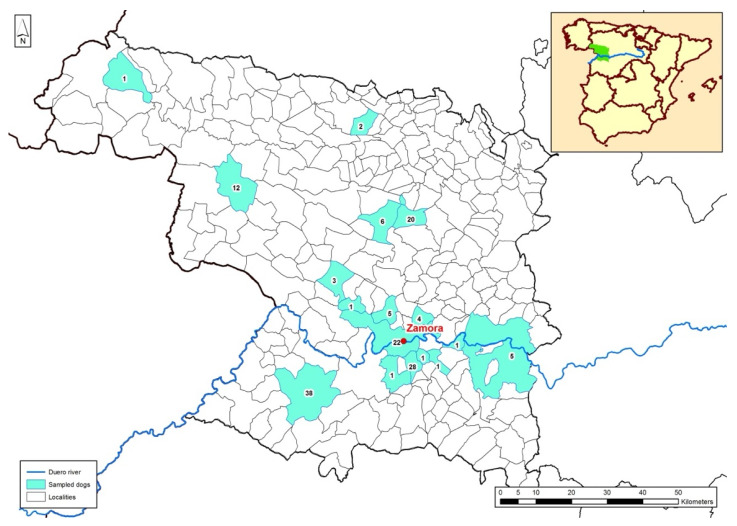
Geographical situation of Zamora province (top right) within the Iberian Peninsula and the distribution and number of samples according to the locality of origin of the dogs.

The study was conducted on 151 apparently healthy dogs of 29 different breeds between 2021 and 2022. Sampling was carried out in 18 localities in the province of Zamora (Figure 1). Zamora is essentially a rural province geographically located in the northwest of Spain and where systematic epidemiological studies on CanL have not been carried out earlier. The province of Zamora has two well-characterised climatic types: a humid climate in the mountainous area (northwest of the province), with abundant rainfall, cold winters, snowfall and mild summers and an extreme continental climate, which affects the rest of the province, with cold winters and hot summers.

In addition to domestic dogs, Zamora has significant canine population used for shepherding and hunting, which has also been sampled in this study. Samples were obtained from dogs undergoing medical evaluation in veterinary centres, or belonging to breeders or hunters. Participation was voluntary. Verbal informed consent was obtained from all owners prior to inclusion in the study. They were fully informed about the objectives of the research, the procedures involved, and the intended use of the data and samples. Nonetheless, the study adhered strictly to ethical principles for animal welfare and good veterinary practice. Inclusion criteria were (i) the absence of previous routine chemoprophylaxis for leishmaniasis and (ii) the owner’s consent to participate in the study.

### 2.2. Sample Collection and Epidemiological Data

The 151 buccal swabs, 1 per dog, were obtained by scraping the mucosa inside the cheeks for at least 1 min. After sample collection, the swabs were immediately sent to the laboratory for qPCR analysis, maintaining the cold chain. Regarding the qPCR-positive animals, all dog owners were informed of the test results. In cases where Leishmania DNA was detected and/or clinical signs suggestive of canine leishmaniosis were observed, appropriate clinical recommendations were made by the attending veterinarians. In most cases, dogs were monitored or treated according to established therapeutic guidelines for leishmaniosis, depending on the individual clinical context and owner decision. No animal was left untreated if clinical signs required intervention.

In addition, epidemiological variables were recorded for each dog on a questionnaire completed by the owner or the veterinarian. These variables were intrinsic factors associated with the dog, its location and/or habitat and possible clinical signs related to CanL (Table 1).

### 2.3. DNA Extraction

The DNA extraction of buccal swabs was performed using the GeneJet Genomic DNA Purification kit (Thermo Scientific, Madrid, Spain), according to the manufacturer’s recommendations. The DNA was stored at −20 °C until use.

### 2.4. Real Time PCR (qPCR)

To obtain total leishmanial DNA, *L. infantum* promastigotes (MON1 zymodeme strain) were routinely cultured in 199 Medium (Gibco Europe, Paisley, UK), supplemented with 10% (*v*/*v*) foetal bovine serum (FBS) (Gibco Europe, Paisley, Scotland, UK) and antibiotic mixture (100 U/mL penicillin and 100 µg/mL streptomycin) (Thermo Scientific, Madrid, Spain) at 26 °C, according to [19]. *L. infantum* promastigotes in log phase of growth were harvested and washed three times by centrifugation at 1500× *g* for 5 min in sterile PBS and counted in a haemocytometer. Genomic DNA was extracted from 200 μL of 26 × 10^6^ promastigotes/mL using the same kit and procedure as described above.

To develop this new qPCR method, a primer pair was designed to amplify a 131-base pair (bp) fragment corresponding to a conserved region of minicircles of kinetoplast DNA (kDNA) from *L. infantum*: (Li_F: 5′-CCCAAACTTTTCTGGTCCTC-3′; Li_R: 5′-TTACACCAACCCCCAGTTTC-3′) [20]. As an internal control, we also quantified the number of canine cells by amplifying a 303 bp fragment of the gene encoding the canine Na^+^/Ca^2+^ exchanger (NCX1) gene: (NCX_F: 5′-CCTAGGTCTCCTGCAGTGAAGT-3′; NCX_R: 5′-CCAAGACCCTTCCTTTGGA-3′) [21].

The amplification was performed in a 7500 Real-Time Applied Biosystem PCR thermocycler. DNA amplification was carried out in 12 μL final volume containing 4 μL total DNA, for amplification of the kDNA minicircle fragment of *L. infantum*, or 1 μL for NCX1 gene amplification, 6 μL of SYBR Premix Ex Taq II Master Mix (Takara Bio Inc., Madrid, Spain), 0.05 μL of Reference Dye II, 0.3 μL of each primer (final concentration: 0.4 μM), and the remaining volume of deionised water (1.35 μL for *L. infantum* qPCR and 4.35 μL for NCX1 qPCR). The amplification conditions were as follows: 95 °C for 30 s, followed by 40 cycles of 95 °C for 5 s and 60 °C for 30 s. A standard curve was generated using serial dilutions of *L. infantum* DNA (described above), ranging from 10^−1^ to 10^−6^. A second standard curve was generated for NCX1 amplification; in this case the gene amplification was standardised with an artificially synthesised target plasmid (303 bp) inserted into the pGEM-T vector (Promega, Madrid, Spain), using serial dilutions of the plasmid from 10^−1^ to 10^−6^. All amplifications were performed in duplicate. Positive and negative controls were included in all reactions to confirm the specificity of the qPCR.

For quantification of parasite load, expressed as *L. infantum* DNA concentration (Li) per 50,000 cells (n Li/50,000 cells), parasite DNA concentration or NCX1 copy number was obtained by plotting the Ct values of each gene against normalised concentration curves. To estimate the number of cells per smear, NCX1 gene copy number was calculated from the initial plasmid concentration [22]. One copy of the gene was considered to be equivalent to one cell. Based on the results, the animals were divided into three ranges of infection: low (0.1–100 Li/50,000 cells), medium (100–1000 Li/50,000 cells) and high (>1000 Li/50,000 cells).

### 2.5. Statistical Analysis

Statistical analysis was performed using the SPSS 22.0 software for Windows (SPSS Inc., Armonk, NC, USA). The Kolmogorov-Smirnov test was carried out to determine if data were normally distributed. The non-parametric Mann-Whitney U test and Kruskal-Wallis test were used to compare continuous variables among different groups. The statistical relationship between the age and weight of dogs with the level of infection was calculated using the non-parametric Spearman’s rank correlation test. Differences were considered significant with a 5% significance level (*p* < 0.05).

## 3. Results

A total of 151 dogs from 18 different localities of Zamora province were sampled during the 2021–2022 period. In the map of Zamora province (Figure 1), the geographical situation and the number of dogs participating in the current study are shown in blue. Total DNA isolated from the oral mucosa of 45 of the 151 dogs sampled in the study amplified to some extent the kDNA of Leishmania minicircles using the primers described in the Section 2, and were therefore considered positive for *L. infantum* infection. So, the prevalence of *L. infantum* infection in the current study population was estimated to be 30%. The qPCR values found in the 45 positive animals ranged from 0.15 Li/50,000 cells to values as high as 10,357.52 Li/50,000 cells. Considering the three ranges of infection described previously, 29 dogs (64.4%) had a low level of infection (0.1–100 Li/50,000 cells), 10 dogs (22.2%) had a moderate level (100–1000 Li/50,000 cells), and 6 dogs (13.3%) had a high level (>1000 Li/50,000 cells).

As expected, most of the dogs sampled showed no clinical signs at CanL and only 10 of the 151 animals (6.6% of the sampled population) showed some clinical signs compatible with *L. infantum* infection. The most frequent clinical signs, in these 10 dogs, were swollen lymph nodes (72.7%) and onychogryphosis (63.6%), followed by areas of alopecia (54.5%), dermatitis (45.5%) and weight loss (45.5%). The least frequent clinical signs were mucosal pallor (27.1%), presence of ulcers (18.2%), epistaxis (9.1%) and ocular lesions (9.1%). All dogs presented more than one clinical sign. After evaluation of the clinical signs and general condition of the animals, 45.5% of the dogs had mild symptomatology and the remaining 54.5% had moderate symptomatology. No dogs with severe symptomatology were found in the study. Remarkably, the 10 dogs presenting clinical signs were positive by qPCR (100%), 3 (30%) with low levels of infection (0.1–100 Li/50,000 cells), 4 (40%) with moderate levels (100–1000 Li/50,000 cells) and the remaining 3 (30%) with high levels (>1000 Li/50,000 cells). The other 35 dogs, despite being positive to some extent for *L. infantum*, did not show any apparent sign CanL and were apparently healthy animals.

Regarding the intrinsic factors inherent to the animal’s nature, the 151 dogs in the sample belonged to a total of 29 breeds. Table 2 shows that with the exception of Warren Hound, one of the breeds from which most samples were collected (25), 10 of which (40%) were positive, there were no breeds of dogs with a particular susceptibility to *L. infantum* CanL. Other animal intrinsic factors that were not statistically related to *L. infantum* infection were weight, sex and hair length, and the latter are shown in Table 3.

Regarding the age of the animals, a positive correlation, r = 0.253 (*p* < 0.01), was observed between the susceptibility of the animals to the disease and the age of the dogs sampled, probably due to a longer exposure to phlebotomine sandfly bites and natural immunosenescence of senior animals. Finally, we thought that the attitude of the sampled dogs could be important for the occurrence of CanL. Due to the rural character of the region sampled, most of the animals included in the study were hunting dogs; however, the percentage of LCan-positive animals was very similar between these and pet dogs. Although there were no significant differences, it was observed that shepherd dogs had a lower percentage of positive animals (9.1%) (Table 3).

The geographical origin of the samples is also shown in Figure 1; a total of 18 localities were sampled. In this case, significant differences in terms of locality of origin and infection level were found (*p* < 0.05). The population of Bermillo de Sayago, placed at the southwest of the province, had the highest number of positive animals. Out of the 38 samples collected at this location, 18 were positive by qPCR, representing 11.92% of the total samples collected, or 40% of all positive samples (Table 4).

In relation to the factors associated with the habitat of the animal, it was observed that there were no differences between the two possible options, whether the animal spent the most time inside the house, or outdoors—living in a kennel. There were also no differences in the level of infection according to the geographical environment in which the dogs lived (urban, peri-urban or rural) or the geographical area of relief, only plains or hills (Table 5). The number of questionnaires included in Table 5 is 148.

## 4. Discussion

Epidemiological data on the distribution of CanL in Spain are not very reliable as not all localities in the Iberian Peninsula have been systematically screened and the diagnostic methods used have been very heterogeneous [23]. Briefly, territories in the Mediterranean basin of Spain are considered endemic, with significantly higher prevalence rates than central-northern areas. In general, in the northern Mediterranean regions, such as Girona, the average prevalence is around 19% [24], while in the southeast of the country, the estimated seroprevalence was 23.7%, and some studies have described rates higher than 40% in localities in the province of Malaga [25]. However, other studies in the Valencian Community and Murcia have shown prevalence rates of approximately 14% [26]. The situation is the opposite in non-Mediterranean northern regions, such as Cantabria, Asturias and Vizcaya, which showed lower prevalence, ranging from 0 to 5%, with some exceptions, such as the province of Ourense, where a prevalence of 35% was reported [15,26]. Actually, in the last two decades, a mean percentage of new seropositive cases of 21.65 was detected in northwestern Spain [27]. On the other hand, the latest seroprevalence data in the region of Castilla y León, where the province of Zamora is located, was 2% [26]. The heterogeneity of these data can be partly attributed to the diversity of techniques and methodologies used in the different studies, which makes accurate quantification of infection difficult. The use of a quantitative method to diagnose for *L. infantum* infections is essential not only to estimate the prevalence of PCR-positive dogs in endemic areas—where a substantial part of the canine population is exposed to the parasite, but only a little fraction manifests clinical symptoms—but also to monitor parasitaemia after treatment [28]. Amplification of minicircle kDNA or the internal transcribed spacer 1 (ITS-1) of ribosomal DNA are the regions of choice for the researchers in detecting the presence of *Leishmania* parasites [28,29,30] in different organic samples [31,32]. In addition, we introduced two innovations in this study, the use of oral mucosal samples obtained by buccal swabs as a minimally invasive alternative that does not require sedation of the animals [33,34], and the quantification of parasite DNA in relation to the canine cells collected in the sample. This innovation allows standardisation of results between samples with different numbers of cells and consequently stratifies the infective loads of animals into different ranges. In addition, it could help veterinarians to select and adapt treatment protocols according to parasite load, thus improving the accuracy of clinical decision-making. The choice of buccal swabs as the biological sample in this study was based on their non-invasive nature, ease of collection and suitability for field conditions. Unlike conventional sampling methods such as blood, lymph node aspirates or bone marrow, oral swabs do not require sedation or specialised handling, and minimise discomfort and risk to the animal. Although more invasive samples may offer higher sensitivity in some clinical settings, buccal swabs represent a practical and ethically advantageous alternative for large-scale screening studies and are especially appropriate when sampling asymptomatic or apparently healthy animals, as was the case in our work.

To our knowledge, no systematic study of canine leishmaniosis has been carried out in Zamora province. The sample of 151 dogs analysed in this study represents approximately 0.52% of the total registered canine population in the province of Zamora (28,915 dogs in 2021; https://www.siacyl.org/, accessed on 23 May 2025). Based on reported CanL prevalence rates in Spain (2–40%), as described above, and assuming an expected prevalence between 2% and 15% for this low-endemic region, we retrospectively evaluated the adequacy of the sample size using the sample size estimation method for proportions in finite populations, based on the binomial distribution with normal approximation (Z-distribution). For this range of expected prevalence, a sample of 31 to 202 dogs would be sufficient with a 95% confidence level and 5% margin of error. Therefore, our sample size falls within an acceptable range and supports the robustness of the unexpectedly high infection rate (30%) observed in this population. Overall, Zamora is a rural inland province in northwest Spain with a humid climate in the mountainous northwest of the province and an extreme continental climate in the rest of the province. Despite the extreme climatic conditions of the region studied, the proliferation of sandflies during the spring and summer seasons demonstrates the adaptability of phlebotomine to a wide range of climatic conditions. Surprisingly, our results revealed an unexpected 30% of qPCR-positive dogs in the sampled territory, highlighting the severity of the situation among domestic canine populations. Such high values support the idea, suggested by other authors [35,36,37], that the disease is likely to be underestimated in inland regions of the country and the need for comprehensive prevalence studies in different geographical regions to know the real distribution of the parasite, which may be aggravated by the impact of global warming on the spread of the vector towards the central-northern areas of the Iberian Peninsula [38]. On the other hand, although no significant human leishmaniasis outbreaks have been reported in the province of Zamora, and official data on human cases remain scarce, the detection of *Leishmania infantum* in a considerable proportion of dogs (30%) suggests a potential risk of zoonotic transmission. This is particularly relevant in rural areas where close interaction between humans, dogs, and sand fly vectors may occur. These findings support the need for integrated surveillance strategies that consider both human and animal health, in line with the One Health approach.

The proficiency of our qPCR method was remarkable, as it was able to detect all symptomatic animals, even those with mild symptoms. The most frequently observed clinical signs were lymph node swelling (72.7%) and onychogryphosis (63.6%), followed by dermatological signs such as alopecia (54.5%), dermatitis (45.5%) and weight loss (45.5%), which are in agreement with those described by other authors [39,40,41].

Regarding intrinsic factors, our results showed that the risk of infection was higher with increasing age of the dogs (r = 0.253), in agreement with several studies [42,43,44,45,46], which could be attributed to the repeated exposure of the animals to *Leishmania* over the time and the decline of the immune system in senior dogs [47]. However, no association was found between the breed of the animals included in the study and the presence of infection, in contrast to previous studies that reported a genetic background of resistance for Ibizan Hound dogs and alleles associated with higher susceptibility to CanL in Boxer dogs, but from which we could not draw any conclusions as only one specimen of the latter breed was qPCR-positive [48].

Another intrinsic factor that may be related to parasite load was animal weight. The lower weight of small dogs is considered a protective factor for the development of infection, possibly due to their smaller body surface area, which makes them less susceptible to sand fly bites [44,45,46,47,48,49]. However, in agreement with other authors, we could not find any statistical relationship between infection and body weight. One possible explanation for this lack of correspondence is that small dogs tend to be from urban environments used to sleeping indoors, which may act as a protective factor [44]. We also found no significant differences with respect to sex of the animals and hair length, in contrast to other authors [43].

As for environmental risk factors, such as whether the animals were predominantly indoors or outdoors, our study fails to detect any significant influence with the level of infection [42,44,49,50]. However, the percentage of positive dogs was almost twice as high in those living outdoors (31.0%) compared to those living indoors (16.7%), possibly due to prolonged exposure to infected phlebotomines in outdoor environments. In our study, there was also no significant relationship between the level of infection and whether the dogs resided predominantly in urban, peri-urban or rural areas. Some research has identified rural and peri-urban settings as a risk factor for dogs, probably due to the proximity of non-domestic animals and disease vectors, as transmission can occur in wild environments [20,43,49,51]. However, other studies have found higher prevalence rates among dogs residing in urban settings [52], whereas other studies have not identified any impact of habitat type [24], in agreement with the results of our work. While both breed and geographical origin were recorded, the sample size and distribution did not allow for a meaningful analysis of correlations between these variables. Some breeds, like hunting dogs, were more common in rural areas with higher infection rates, but no clear breed-location pattern can be established from the current data. Finally, the attitude given to the dogs may thus play a role in the susceptibility to the disease. According to our results, the prevalence of CanL in hunting dogs was higher than in domestic or herding dogs, which may be explained by the lifestyle of these animals with prolonged stays outdoors and therefore higher exposure to sandflies [24,42,43,44].

## 5. Conclusions

This study provides new molecular evidence of *Leishmania infantum* infection in dogs from the province of Zamora, in northwestern Spain—a region traditionally considered to have low endemicity and limited available data. The use of a non-invasive sampling method (buccal swabs) combined with qPCR detection allowed for the identification and quantification of the parasite in a significant number of animals, including asymptomatic cases. The detection of a considerable proportion of PCR-positive dogs highlights the potential relevance of this area in the current epidemiological landscape of canine leishmaniosis. These findings support the usefulness of molecular tools for surveillance and encourage the implementation of broader epidemiological studies to better understand the distribution and dynamics of infection in inland regions of Spain.

## Figures and Tables

**Table 1:** The epidemiological variables collected on a questionnaire.

Factors associated with the dog	Age	Months
	Hair length	Short: 1; Medium: 2; Long: 3
	Sex	Male or Female
	Weight	kg
	Breed	Name of the breed
	Aptitude	Company: 1; Shepherd: 2; Hunting: 3
Factors associated with location	Sampling location	Name of the location
	Most frequent habitat	Inside a house: 1; Outdoors (in a kennel): 2
	Geographical environment	Urban: 1; Peri-urban: 2; Rural: 3
	Geographical area relief	Plain: 1; Hill: 2; Mountain: 3
	Overnight stay	Inside: 1; Outside: 2
Factors associated with possible clinical signs related to leishmaniasis	Presence or absence	Yes/No
	Type of clinical signs	Swollen lymph nodes: 1
		Dermatitis: 2
		Alopecia: 3
		Ulcers: 4
		Onychogryphosis: 5
		Epistaxis: 6
		Mucous membranes pallor: 7
		Ocular lesions: 8
		Weight loss: 9
	Intensity of clinical signs	Mild: 1; Moderate: 2; Severe: 3

**Table 2:** Number of samples (N) tested by breed and positive results by qPCR.

Dog Breed	N	Positive by qPCR
Spanish Alano	2	1
Blue Griffon of Gascony	4	2
Brittany Fawn Basset	2	1
Beagle	2	-
Bichon Maltes	3	1
Andalusian Buzzard	2	1
Boxer	1	1
German Barco	1	-
French Bulldog	1	-
Deutsch Drahthaar	1	-
German Pug or Great Dane	1	-
Epagneul Breton	21	8
Fox Terrier	1	-
Spanish Galgo	13	2
Golden Retriever	4	3
Spanish Mastin	5	1
Crossbreed or Mongrel Dog	23	6
Pachon Navarro Dog	1	-
German Sheperd	1	-
Brie Shepherd	1	-
Burgos Retriever	2	-
Petit Basset	1	-
Warren Hound	25	10
Spanish Bloodhound	3	1
Miniature Schnauzer	2	-
English Setter	26	7
Irish Setter	2	-

**Table 3:** Intrinsic factors associated with the dog. N: number of dogs; Positive: number of positive 205 dogs by qPCR and its corresponding percentage.

Variable		N	Positive (%)		N	Positive (%)		N	Positive (%)
Sex	Male	80	27 (33.8%)	Female	71	16 (22.5%)	-	-	-
Hair length	Short	66	21 (31.8%)	Medium	47	13 (27.7%)	Long	38	11 (28.9%)
Aptitude	Company	17	6 (35.3%)	Shepherd	11	1 (9.1%)	Hunting	123	38 (30.9%)

**Table 4:** Number of samples (N) tested by location and positive results by qPCR.

Population	Samples Collected	Positive by PCR	Percentage (%)
Arcenillas	1	0	-
Bermillo de Sayago	38	18	11.92
Carbajales de Alba	3	0	-
Zamora	15	0	-
Carrascal	7	1	0.6
Granja de Moreruela	20	4	2.6
Granucillo de Vidriales	2	0	-
La Inhiesta	5	0	-
Monfarracinos	4	3	1.99
Moraleja del Vino	1	0	-
Morales del Vino	28	10	6.62
El Perdigón	1	1	0.6
Pobladura de Aliste	12	5	3.31
Ribadelago	1	0	-
San Pedro de la Nave	1	0	-
Santa Eulalia de Tábara	6	3	1.99
Toro	5	0	-
Villalazán	1	0	
Total	151	45	29.8

**Table 5:** Factors associated with location. N: number of dogs; Positive: number of positive dogs by qPCR and its corresponding percentage.

Variable		N	Positive (%)		N	Positive (%)		N	Positive (%)
Most frequent habitat during the day	Inside a house	6	1 (16.7%)	Outdoors	142	44 (31.0%)	-	-	-
Geographical environment	Urban	6	2 (33.3%)	Peri-urban	59	13 (22.0%)	Rural	83	30 (36.1%)
Geographical area relief	Plain	109	36 (33.0%)	Hill	39	9 (23.1%)	Mountain	0	0 (0.00%)
Overnight stay	Inside	138	42 (30.4%)	Outside	11	3 (27.3%)	-	-	-

## Data Availability

The raw data supporting the conclusions of this article will be made available by the authors on request.
